# Reverse complete heart block using transcutaneous pacing and repeated plasmapheresis in a neonate with lupus: a case report

**DOI:** 10.1186/s12969-023-00920-w

**Published:** 2023-11-09

**Authors:** Yanfei Liu, Wanwei Li, Kun Zhou, Zhangxue Hu

**Affiliations:** Department of Pediatrics & Neonatology, Army Medical Center, Army Medical University, Chongqing, 400042 China

**Keywords:** Neonatal systemic Lupus Erythematosus, Congenital heart block, Cardiac pacing, Artificial, Plasmapheresis, Case report

## Abstract

**Background:**

It has been reported that the complete heart block (CHB) in neonatal lupus (NL) cannot be reversed. This study reported a case of NL-CHB that was reversed by transcutaneous pacing and repeated plasmapheresis.

**Case presentation:**

A 35^+ 6^-week male preterm baby was transferred to the neonatal intensive care unit of the Army Medical Center in May 2020 for slight cyanosis around the lips and nose. Two days after birth, a sudden decrease in heart rate was observed during electrocardiogram (EGG) monitoring. Physical examination revealed a bluish-purple discoloration around the lips and an irregular heartbeat. EGG showed the presence of isolated P (142 bpm) and QRS (78 bpm) waves, ventricular escape beats, and a diagnosis of NL-CHB. To reverse the condition, transcutaneous pacing and five sessions of plasmapheresis were performed. At a 1.5-year follow-up, the baby exhibited well-developed cardiac structure and normal neurodevelopment.

**Conclusions:**

Transcutaneous pacing and repeated plasmapheresis might be possible to reverse CHB in NL.

**Supplementary Information:**

The online version contains supplementary material available at 10.1186/s12969-023-00920-w.

## Background

Nearly 1% of all pregnancies are complicated by maternal autoimmune disease, which include systemic lupus erythematosus (SLE), Sjögren syndrome, rheumatoid arthritis, mixed connective tissue disease, an undifferentiated autoimmune disease, or neonatal lupus (NL) with cutaneous and/or cardiac manifestations in a previous pregnancy. The passive passage of autoantibodies from the mother will result in NL in 2-3% of the fetuses, which is associated with cardiac and cutaneous complications [1]. The III° atrioventricular block (AVB), also named complete heart block (CHB), significantly reduces the neonates’ cardiac output for low heart rate and has a mortality of over 20% despite pacemaker implantation (PMI) and much higher without PMI, and most of the patients die of acute heart failure and/or dilated cardiomyopathy (DCM) [2]. PMI is recommended for all neonatal lupus-complete heart block (NL-CHB) patients because CHB is considered irreversible in the current opinions [3]. Here, this study reported a case of NL-CHB that was reversed by transcutaneous pacing and repeated plasmapheresis.

## Case presentation

**Fig. 1:**
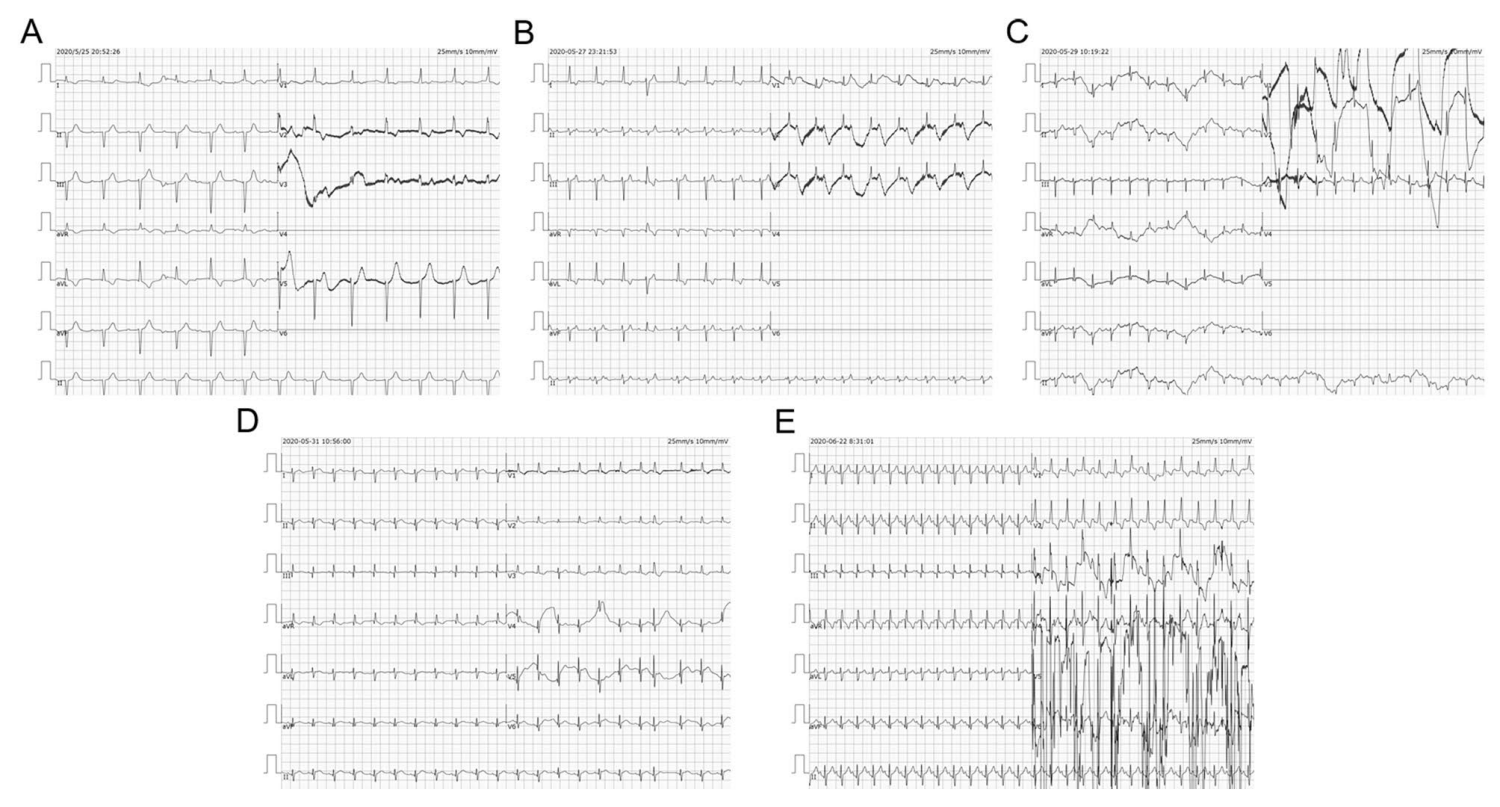
ECG records before and after plasmapheresis. **A** (3 days after birth) the first EEG on the beginning of AVB. P(142 bpm) and QRS (78 bpm) were isolated, ventricular escape beat, incomplete right bundle branch block, with ST-T change. **B** (5 days after birth) Sinus rhythm, ectopic rhythm and IIIAVB. **C** (7 days after birth) Sinus rhythm, frequent Ventricular premature beat. **D** (9 days after birth) Sinus rhythm and occasional atrial premature beats. **E** Sinus rhythm

A preterm male baby (gestational age of 35^+ 6^ weeks, body weight 2.74 kg) was transferred to the neonatal intensive care unit (NICU) of the Army Medical Center in May 2020 for slight cyanosis around the lips and nose. The baby was delivered by C-section for intrauterine stress, and the second birth of a mother with psoriasis and severe intrahepatic cholestasis. The mother had regular antenatal care including the fetal heart monitoring, but without treatment for her ten-year psoriasis. Meanwhile, the fetal bradycardia was not detected during the antenatal follow-up. Prior to delivery, The mother’s serum antibodies were ANA (1:1000), SSA (+++), SSB (+++), and Ro-52 (+++). The baby’s myocardial enzyme spectrum after admission indicated an increasing in creatine kinase-myocardial band isoenzyme (CK-MB). Electrocardiogram (ECG) monitoring showed a sudden onset of heart rate fall, P (142 bpm) and QRS (78 bpm) were isolated (IIIAVB), ventricular escape beat, incomplete right bundle branch block, with ST-T change at 3 days after birth **(**Fig. 1A**)**. Addtionally, deoxygenation [oxygen saturation (SpO_2_) < 80%] with severe myocardial injury and acute heart failure [brain natriuretic peptide (BNP) > 5000 pg/mL] were observed immediately and persisted. Physical examination showed a bluish-purple coloration around the lips, a red rash on the anterior chest, irregular heartbeat with weak and uneven heart sounds, and blood pressure of 76/44 mmHg. Ultrasound showed generalized cardiac enlargement, severe mitral valve regurgitation, severe tricuspid regurgitation, pulmonary artery hypertension, bradycardia, and segregation of atrial septal anomalies. ECG showed sinus rhythm, ectopic rhythm,and IIIAVB. **(**Fig. 1B**)**. Chest X-ray showed generalized cardiac enlargement. The immunofluorescence density of SSA, SSB, and RO-52 antibodies was strongly positive, and ANA was 1:320. Brain magnetic resonance imaging (MRI) showed white matter injury (WMI) under the right side of the parietal lobe, with a significant elevation of tumor necrosis factor (TNF)-α and interleukin (IL)-6. CHB-NL, heart failure, and dilated cardiomyopathy was diagnosed.

The antiarrhythmic drugs isoproterenol and adrenaline used to increase the ventricular rate and enhance cardiac output in the early stage (< 24 h) soon became ineffective, and intractable heart failure led to agonal stage signs 24 h after the break. Transcutaneous pacing was performed using a defibrillator (M series, ZOLL Medical Corporation, Chelmsford, MA, USA) with the pace function module with a pace current of 30 mA and pace heart rate (HR) of 120 bpm. Plasmapheresis and hormone shock therapy were performed. After the first plasmapheresis session, ECG monitoring showed sinus rhythm, frequent Ventricular premature beat **(**Fig. 1C**)**. The second and third plasmapheresis sessions reversed the CHB back to occasional atrial premature beats **(**Fig. 1D**)**. When a new complete right bundle branch block was detected, two partial plasmapheresis sessions were performed, and the HR returned to alternate incomplete right bundle branch block or normal. After 1 month of birth, the patient was discharged from the hospital with an HR of 130–148 bpm **(**Fig. 1E**)** and the antibody titers were SSA (+++), SSB (+), and Ro-52 (+++) **(**Table 1**)**. At 1-year follow-up after birth, growth and development were normal. SSA, SSB, and Ro-52 were negative. MRI showed no significant changes in the softening lesion in the right parietal lobe. The baby had grown up healthy to 1.5 years, with a well-developed cardiac structure and normal neurodevelopment scores.

**Table 1:** Characteristics of the patient in time

Parameters	D1	D3	D4	D5	D6	D7	D8	D9	D10	M1	M2	M6
ECG	NSR	III° AVB	III° AVB	III° AVB	NSR	NSR	NSR	NSR	NSR	NSR	NSR	NSR
Lowest HR (bpm)	144	65	70	60	100	100	110	112	108	110	115	112
Lowest BP (mmHg)	78/50	76/44	55/28	60/41	80/58	88/50	94/59	89/52	87/49	90/50	93/52	91/49
Lowest SpO_2_ (%)	95	80	50	70	97	92	95	93	94	93	95	96
Lowest PaO_2_ (mmHg)	80	58	38	46	74	82	69	71	56	82	85	80
SSA	/	/	+++	+++	+++	+++	+++	+++	+++	+++	++	+
SSB	/	/	+++	+++	+++	+++	+++	+++	+++	++	+	+
RO52	/	/	+++	++	++	++	++	++	++	++	+	+
ANA	/	/	1:320	1:100	1:100	1:100	1:100	1:100	1:100			
BNP (pg/ml)	/	> 5000	> 4998	> 5000	> 5000	> 5000	1109.39	235.8	180.5	100.5	21.82	20.05
CK-MB (µg/L)	/	34.52	/	/	/	/	0.88	/	/	6.42	/	/
Myoglobin (µg/L)	/	135.2	/	/	/	/	< 21	/	/	< 21	/	/
TnT-HSST (µg/L)	/	4.48	/	/	/	/	0.037	/	/	0.094	/	/
CK (U/L)	450.4	/	/	129.64	/	/	/	/	140.28	120.7	72.9	/
LVDd (mm)	/	15	19	19	/	18	/	17	17	16	17	20
LVEF (%)	/	68	64	45	/	66	/	70	65	68	69	65
PAP (mmHg)	/	19	53	41	/	22	/	22	26	20	25	
WBC (×10^9^/L)	7.62	11.19	14.93	14.06	7.98	9.29	8.63	8.25	7.68	7.95	8.23	9.13
RBC (×10^12^/L)	5.67	3.31	3.58	14.62	3.18	4.22	4.05	4.11	4.05	3.85	4.13	4.66
HGB (g/L)	217	121	126	105	101	136	131	128	126	125	112	130
PLT (×10^6^/L)	198	148	146	181	138	162	116	124	132	158	163	195
PCT (ng/ml)	0.12	1.4	/	/	/	0.17	0.07	0.06	0.05	0.05	0.03	/
CRP (mg/L)	< 0.5	0.86	1.79	1.34	0.49	< 0.5	< 0.5	< 0.5	< 0.5	< 0.5	< 0.5	< 0.5
C3 (g/l)	/	0.49	/	0.55	0.54	/	0.68	/	/	0.90	0.84	0.81
C4 (g/l)	/	0.13	/	0.12	0.15	/	0.14	/	/	0.19	0.17	0.20
pH	7.493	7.48	7.45	7.43	7.42	7.49	7.38	7.39	7.38	7.40	7.42	7.44
PO_2_ (mmHg)	83	38	34	37	126	71	56	86	92	95	102	88
Lac (mmol/L)	/	8.2	5.1	7.3	7.0	4.5	2.3	2.1	1.8	1.0	0	0

D1-D10: day of birth; M1-M6: months of birth; ECG: electrocardiogram; NSR: normal sinus rhythm; AVB: atrial ventricular block; HR: heart rate; BP: blood pressure; SpO_2_: oxygen saturation; PaO_2_: partial pressure of oxygen; ANA: antinuclear antibody; BNP: brain natriuretic peptide; CK-MB: creatinine kinase MB; TnT-HSST: troponin T-hypersensitivity; CK: creatinine kinase; LVDd: left ventricle end-diastolic diameter; LVEF: left ventricle ejection fraction: PAP: pulmonary artery pressure; WBC: white blood cells; RBC: red blood cells; HGB: hemoglobin; PLT: platelets; PCT: procalcitonin; CRP: C-reactive protein; C3: complement 3; C4; complement 4; PO_2_: partial pressure of oxygen; Lac: lactate; /: missing; -: negative

## Discussion and conclusions

This report presents a case of NL-CHB that was reversed by transcutaneous pacing and repeated plasmapheresis, suggesting that reversing CHB might be possible in some NL cases.

Although previous studies suggested the use of fluorinated steroids [4] and maternal plasmapheresis [5] during pregnancy in women with known autoimmune diseases, both failed in preventing NL-related CHB. The CHB has a specific affinity to the SSA/SSB/Ro-52 antibodies, leading to sustained immune attack till entire fibrosis-induced irreversible AVB dysfunction [6]. Nevertheless, a study reported a time window from the initial antibody attack to CHB permanent damage, and the dysfunction might persist for several days despite ongoing resolution [7]. Therefore, quickly eliminating the SSA/SSB/Ro-52 antibodies in the time window can provide a chance to rescue the CHB and rebuild sinus rhythm [8], at the condition that the initial antibody attack occurred not too long before birth and the neonate is still in the actionable window. Since the antibodies found in the neonate come, in fact, from the mother and are not produced by the neonate, performing plasmapheresis on the baby instead of the mother might be more effective, as maternal plasmapheresis appears unsuccessful [5], while it was successful in the case presented here. Reports on the use of plasmapheresis in neonates are extremely scarce, reflecting the technical difficulties and potential risks in carrying out this procedure in this age group.This forefront treatment is effectively used to treat severe conditions in neonates, such as neonatal hyperbilirubinemia [9, 10], acute kidney injury [11], and Hemolytic Uremic Syndrome [12]. Sawyer et al. [13] examined the outcomes of a cohort of neonates with septic shock treated with plasmapheresis, and found that plasmapheresis can be performed in critically ill neonates with severe septic shock on extracorporeal life support and may be beneficial in select cases. LiKamWa et al. [14] have reported that a neonate with cytokine storm managed with plasmapheresis, steroids, and Tocilizumab, resulting in stable discharge of the baby. But to the best of our knowledge, the neonate described here is the first reported patient with NL-CHB to undergo plasmapheresis therapy. The efforts to reverse CHB back to normal sinus rhythm (NSR) should continue because lifelong pacemaker implantation brings potential risk in childhood and adult.

A study demonstrated that anti-Ro/SSA antibodies can cause brain injury in neonates [15]. NL-related brain injury has become an important hot topic for possible neurodevelopmental issues. Brain injury was detected in this case by MRI (with diffusion tensor imaging data). Although neurodevelopment evaluation for this baby showed a normal score in early life (1.5 years), long-term follow-up is needed. Thus, although a quick anti-Ro/SSA antibody clearance reverses CHB, whether it could also avoid neural impairment remains to be determined.

Another important treatment for this case was the application of transcutaneous pacing, which stabilized the cardiac output and opened an opportunity for plasmapheresis. Temporary pacing is particularly helpful in patients with reversible or transient conditions or when transvenous pacing is not immediately available or possible. Very limited experience from reported cases showed that pace could be performed in neonates [16]. In this case, transcutaneous pacing showed a rapid and stable effect.

In conclusion, transcutaneous pacing and repeated plasmapheresis might be possible to reverse CHB in NL using.

### Electronic supplementary material

Below is the link to the electronic supplementary material.

Supplementary Material 1

Supplementary Material 2

## Data Availability

All data generated or analysed during this study are included in this published article.

## Abbreviations

NL: Neonatal lupus
AVB: Atrial-ventricle block
CHB: Complete heart block
NL-CHB: Neonatal lupus-complete heart block
PMI: Permanent pacemaker implantation
NSR: Normal sinus rhythm
DCM: Dilated cardiomyopathy
ECG: Electrocardiogram
DTI: Diffusion tensor imaging
