# Supplementary material for: Reverse complete heart block using transcutaneous pacing and repeated plasmapheresis in a neonate with lupus: a case report
Source: Pediatr Rheumatol Online J. 2023 Nov 9;21:135. doi: 10.1186/s12969-023-00920-w (PMC10636865; doi:10.1186/s12969-023-00920-w)
Supplement: Supplementary file 1 — Supplementary Material 1 [file 12969_2023_920_MOESM1_ESM.docx]

**Reverse complete heart block using transcutaneous pacing and repeated plasmapheresis in a neonate with lupus: A case report**

**Running title: Reverse CHB in a NL**

Yanfei Liu^#^, Wanwei Li^#^, Kun Zhou^#^, Zhangxue Hu*,

Department of Pediatrics & Neonatology, Army Medical Center, Army Medical University, Chongqing, China, 400042

^#^These authors contributed equally to this work.

**^*^Corresponding author:**

Prof. Zhangxue Hu,

Department of Pediatrics & Neonatology, Army Medical Center, Army Medical University, Chongqing, China, 400042

No.10 Changjiangzhilu, Yuzhong district, Chongqing, China, 400042

Email: [huzx1@163.com](mailto:huzx1@163.com)

Tel: 8618696668598

**Abstract**

**Background:** It has been reported that the complete heart block (CHB) in neonatal lupus (NL) cannot be reversed. This study reported a case of NL-CHB that was reversed by transcutaneous pacing and repeated plasmapheresis.

**Case presentation:** A 35^+6^-week male preterm baby was transferred to the neonatal intensive care unit of the Army Medical Center in May 2020 for slight cyanosis around the lips and nose. Two days after birth, a sudden decrease in heart rate was observed during electrocardiogram (EGG) monitoring. Physical examination revealed a bluish-purple discoloration around the lips and an irregular heartbeat. EGG showed the presence of isolated P (142 bpm) and QRS (78 bpm) waves, ventricular escape beats, and a diagnosis of NL-CHB. To reverse the condition, transcutaneous pacing and five sessions of plasmapheresis were performed. At a 1.5-year follow-up, the baby exhibited well-developed cardiac structure and normal neurodevelopment.

**Conclusions:** Transcutaneous pacing and repeated plasmapheresis might be possible to reverse CHB in NL.

**Keywords:** Neonatal systemic lupus erythematosus; Congenital heart block; Cardiac Pacing, Artificial; Plasmapheresis; Case report

**Background**

Nearly 1% of all pregnancies are complicated by maternal autoimmune disease, which include systemic lupus erythematosus (SLE), Sjögren syndrome, rheumatoid arthritis, mixed connective tissue disease, an undifferentiated autoimmune disease, or neonatal lupus (NL) with cutaneous and/or cardiac manifestations in a previous pregnancy. The passive passage of autoantibodies from the mother will result in NL in 2%-3% of the fetuses, which is associated with cardiac and cutaneous complications (1). The Ⅲ° atrioventricular block (AVB), also named complete heart block (CHB), significantly reduces the neonates’ cardiac output for low heart rate and has a mortality of over 20% despite pacemaker implantation (PMI) and much higher without PMI, and most of the patients die of acute heart failure and/or dilated cardiomyopathy (DCM) (2). PMI is recommended for all neonatal lupus-complete heart block (NL-CHB) patients because CHB is considered irreversible in the current opinions (3). Here, this study reported a case of NL-CHB that was reversed by transcutaneous pacing and repeated plasmapheresis.

**Case presentation**

A preterm male baby (gestational age of 35^+6^ weeks, body weight 2.74 kg) was transferred to the neonatal intensive care unit (NICU) of the Army Medical Center in May 2020 for slight cyanosis around the lips and nose. The baby was delivered by C-section for intrauterine stress, and the second birth of a mother with psoriasis and severe intrahepatic cholestasis. The mother had regular antenatal care including the fetal heart monitoring, but without treatment for her ten-year psoriasis. Meanwhile, the fetal bradycardia was not detected during the antenatal follow-up. Prior to delivery, The mother’s serum antibodies were ANA (1:1000), SSA (+++), SSB (+++), and Ro-52 (+++). The baby’s myocardial enzyme spectrum after admission indicated an increasing in creatine kinase-myocardial band isoenzyme (CK-MB). Electrocardiogram (ECG) monitoring showed a sudden onset of heart rate fall, P (142bpm) and QRS (78bpm) were isolated (ⅢAVB), ventricular escape beat, incomplete right bundle branch block, with ST-T change at 3 days after birth **(Figure 1A)**. Addtionally, deoxygenation [oxygen saturation (SpO_2_) <80%] with severe myocardial injury and acute heart failure [brain natriuretic peptide (BNP) >5000 pg/mL] were observed immediately and persisted. Physical examination showed a bluish-purple coloration around the lips, a red rash on the anterior chest, irregular heartbeat with weak and uneven heart sounds, and blood pressure of 76/44 mmHg. Ultrasound showed generalized cardiac enlargement, severe mitral valve regurgitation, severe tricuspid regurgitation, pulmonary artery hypertension, bradycardia, and segregation of atrial septal anomalies. ECG showed sinus rhythm, ectopic rhythm,and ⅢAVB. **(Figure 1B)**. Chest X-ray showed generalized cardiac enlargement. The immunofluorescence density of SSA, SSB, and RO-52 antibodies was strongly positive, and ANA was 1:320. Brain magnetic resonance imaging (MRI) showed white matter injury (WMI) under the right side of the parietal lobe, with a significant elevation of tumor necrosis factor (TNF)-α and interleukin (IL)-6. CHB-NL, heart failure, and dilated cardiomyopathy was diagnosed.

The antiarrhythmic drugs isoproterenol and adrenaline used to increase the ventricular rate and enhance cardiac output in the early stage (<24 h) soon became ineffective, and intractable heart failure led to agonal stage signs 24 h after the break. Transcutaneous pacing was performed using a defibrillator (M series, ZOLL Medical Corporation, Chelmsford, MA, USA) with the pace function module with a pace current of 30 mA and pace heart rate (HR) of 120 bpm. Plasmapheresis and hormone shock therapy were performed. After the first plasmapheresis session, ECG monitoring showed sinus rhythm, frequent Ventricular premature beat **(Figure 1C)**. The second and third plasmapheresis sessions reversed the CHB back to occasional atrial premature beats **(Figure 1D)**. When a new complete right bundle branch block was detected, two partial plasmapheresis sessions were performed, and the HR returned to alternate incomplete right bundle branch block or normal. After 1 month of birth, the patient was discharged from the hospital with an HR of 130-148 bpm **(Figure 1E)** and the antibody titers were SSA (+++), SSB (+), and Ro-52 (+++) **(Table 1)**. At 1-year follow-up after birth, growth and development were normal. SSA, SSB, and Ro-52 were negative. MRI showed no significant changes in the softening lesion in the right parietal lobe. The baby had grown up healthy to 1.5 years, with a well-developed cardiac structure and normal neurodevelopment scores.

**Discussion and Conclusions**

This report presents a case of NL-CHB that was reversed by transcutaneous pacing and repeated plasmapheresis, suggesting that reversing CHB might be possible in some NL cases.

Although previous studies suggested the use of fluorinated steroids (4) and maternal plasmapheresis (5) during pregnancy in women with known autoimmune diseases, both failed in preventing NL-related CHB. The CHB has a specific affinity to the SSA/SSB/Ro-52 antibodies, leading to sustained immune attack till entire fibrosis-induced irreversible AVB dysfunction (6). Nevertheless, a study reported a time window from the initial antibody attack to CHB permanent damage, and the dysfunction might persist for several days despite ongoing resolution (7). Therefore, quickly eliminating the SSA/SSB/Ro-52 antibodies in the time window can provide a chance to rescue the CHB and rebuild sinus rhythm (8), at the condition that the initial antibody attack occurred not too long before birth and the neonate is still in the actionable window. Since the antibodies found in the neonate come, in fact, from the mother and are not produced by the neonate, performing plasmapheresis on the baby instead of the mother might be more effective, as maternal plasmapheresis appears unsuccessful (5), while it was successful in the case presented here. Reports on the use of plasmapheresis in neonates are extremely scarce, reflecting the technical difficulties and potential risks in carrying out this procedure in this age group.This forefront treatment is effectively used to treat severe conditions in neonates, such as neonatal hyperbilirubinemia (9,10), acute kidney injury (11), and Hemolytic Uremic Syndrome (12). Sawyer et al. (13) examined the outcomes of a cohort of neonates with septic shock treated with plasmapheresis, and found that plasmapheresis can be performed in critically ill neonates with severe septic shock on extracorporeal life support and may be beneficial in select cases. LiKamWa et al. (14) have reported that a neonate with cytokine storm managed with plasmapheresis, steroids, and Tocilizumab, resulting in stable discharge of the baby. But to the best of our knowledge, the neonate described here is the first reported patient with NL-CHB to undergo plasmapheresis therapy. The efforts to reverse CHB back to normal sinus rhythm (NSR) should continue because lifelong pacemaker implantation brings potential risk in childhood and adult.

A study demonstrated that anti-Ro/SSA antibodies can cause brain injury in neonates (15). NL-related brain injury has become an important hot topic for possible neurodevelopmental issues. Brain injury was detected in this case by MRI (with diffusion tensor imaging data). Although neurodevelopment evaluation for this baby showed a normal score in early life (1.5 years), long-term follow-up is needed. Thus, although a quick anti-Ro/SSA antibody clearance reverses CHB, whether it could also avoid neural impairment remains to be determined.

Another important treatment for this case was the application of transcutaneous pacing, which stabilized the cardiac output and opened an opportunity for plasmapheresis. Temporary pacing is particularly helpful in patients with reversible or transient conditions or when transvenous pacing is not immediately available or possible. Very limited experience from reported cases showed that pace could be performed in neonates (16). In this case, transcutaneous pacing showed a rapid and stable effect.

In conclusion, transcutaneous pacing and repeated plasmapheresis might be possible to reverse CHB in NL using.

**List of abbreviations**

NL: neonatal lupus,

AVB: atrial-ventricle block,

CHB: complete heart block,

NL-CHB: neonatal lupus-complete heart block

PMI: permanent pacemaker implantation,

NSR: normal sinus rhythm,

DCM: dilated cardiomyopathy,

ECG: electrocardiogram,

DTI: diffusion tensor imaging

**Declarations**

**Ethics approval and consent to participate**

All procedures were performed in accordance with the ethical standards laid down in the 1964 Declaration of Helsinki and its later amendments. This study was approved by the Ethics Committee of Army Medical Center [Medical Research Ethics (2023) No. 192], All patients provided written informed consent prior to treatment. All methods were carried out in accordance with relevant guidelines and regulations.

**Consent for publication**

Not applicable

**Availability of data and materials**

All data generated or analysed during this study are included in this published article.

**Competing interests**

The authors have no conflicts of interest relevant to this article to disclose.

**Funding**

National key research project for women and children health of China (No. 2021YFC2701700), funding the gene tests for the family, the publication fees of open access, and consultation with national specialists cooperated in the rescue process.

**Authors' contributions**

H ZX was responsible for all treatments, drafted the initial manuscript, and reviewed and revised the manuscript.
L YF, L WW, and Z K discussed the case, performed planned therapies, collected data, carried out the initial analyses, and reviewed and revised the manuscript.
All authors approved the final manuscript as submitted and agree to be accountable for all aspects of the work.

**Acknowledgement**

Prof. Hu Yijie from the Department of cardiac surgery conceptualized and designed the transcutaneous pacing. Prof. Yang Jie and his team from the Department of Nephrology performed the plasmapheresis. Prof. Fang Yuqiang from the Department of Cardiology analyzed all ECGs and confirmed AVB diagnosis. Mrs. Liu Shu collected clinical data and images during draft preparation. Mrs. Zhang Li and Mrs. Ran Xin did great work in nursing the baby during the rescue procedure. Mrs. Li Mengchun performed most treatments as chief resident in the NICU.

**References**

1. Diaz-Frias J, Badri T. Neonatal Lupus Erythematosus. StatPearls. Treasure Island (FL) ineligible companies. Disclosure: Talel Badri declares no relevant financial relationships with ineligible companies.2023.

2. Tsujii N, Miyazaki A, Sakaguchi H, Kagisaki K, Yamamoto T, Matsuoka M, et al. High Incidence of Dilated Cardiomyopathy After Right Ventricular Inlet Pacing in Patients With Congenital Complete Atrioventricular Block. Circ J. 2016;80(5):1251-8.

3. Kikano SD, Killen SAS. Transient fetal atrioventricular block: A series of four cases and approach to management. J Cardiovasc Electrophysiol. 2022;33(10):2228-32.

4. Izmirly PM, Saxena A, Sahl SK, Shah U, Friedman DM, Kim MY, et al. Assessment of fluorinated steroids to avert progression and mortality in anti-SSA/Ro-associated cardiac injury limited to the fetal conduction system. Ann Rheum Dis. 2016;75(6):1161-5.

5. Miyakata S, Takeuchi K, Yamaji K, Kanai Y, Tsuda H, Takasaki Y. [Therapeutic plasmapheresis for the prevention of congenital complete heart block associated with anti-SS-A/Ro antibody and anti-SS-B/La antibody]. Ryumachi. 2001;41(4):726-35.

6. Clancy RM, Neufing PJ, Zheng P, O'Mahony M, Nimmerjahn F, Gordon TP, et al. Impaired clearance of apoptotic cardiocytes is linked to anti-SSA/Ro and -SSB/La antibodies in the pathogenesis of congenital heart block. J Clin Invest. 2006;116(9):2413-22.

7. Cuneo BF, Sonesson SE, Levasseur S, Moon-Grady AJ, Krishnan A, Donofrio MT, et al. Home Monitoring for Fetal Heart Rhythm During Anti-Ro Pregnancies. J Am Coll Cardiol. 2018;72(16):1940-51.

8. Tonello M, Ruffatti A, Marson P, Tison T, Marozio L, Hoxha A, et al. Plasma exchange effectively removes 52- and 60-kDa anti-Ro/SSA and anti-La/SSB antibodies in pregnant women with congenital heart block. Transfusion. 2015;55(7):1782-6.

9. Wang JF, Ma L, Gong XH, Cai C, Sun JJ. Severe hyperbilirubinemia in a neonate with hereditary spherocytosis due to a de novo ankyrin mutation: A case report. World J Clin Cases. 2021;9(19):5245-51.

10. Belousova T, Tong Y, Bai Y, Klein K, Tint H, Castillo B. Utilization of therapeutic plasma exchange for hyperbilirubinemia in a premature newborn on extracorporeal membrane oxygenation. J Clin Apher. 2019 Oct;34(5):615-22.

11. Tufan Pekkucuksen N, Sigler KE, Akcan Arikan A, Srivaths P. Tandem plasmapheresis and continuous kidney replacement treatment in pediatric patients. Pediatr Nephrol. 2021;36(5):1273-8.

12. Almoshary MA, Alswyeh R, Edrees BM. Successful Treatment of Atypical Hemolytic Uremic Syndrome With Therapeutic Plasma Exchange in a 3.8-kg Neonate. Ther Apher Dial. 2017 Apr;21(2):207-8.

13. Sawyer T, Billimoria Z, Handley S, Smith K, Yalon L, Brogan TV, DiGeronimo R. Therapeutic Plasma Exchange in Neonatal Septic Shock: A Retrospective Cohort Study. Am J Perinatol. 2020;37(9):962-9.

14. LiKamWa A, Kobaitri K, Totapally BR. A Neonate With Cytokine Storm Managed With Steroids, Therapeutic Plasma Exchange, and Tocilizumab. Cureus. 2023;15(9):e45138.

15. Wang XG, Hao QF, Cheng XY. [Neonatal lupus complicated by stroke: case report]. Zhonghua Er Ke Za Zhi. 2016;54(12):952-3.

16. Khanna P, Arora S, Aravindan A, Prasad G. Anesthetic management of a 2-day-old with complete congenital heart block. Saudi J Anaesth. 2014;8(1):134-7.

**Figure Legends**

**Figure 1 ECG records before and after plasmapheresis.** A (3 days after birth) the first EEG on the beginning of AVB. P(142bpm) and QRS (78bpm) were isolated, ventricular escape beat, incomplete right bundle branch block, with ST-T change. B (5 days after birth) Sinus rhythm, ectopic rhythm and ⅢAVB. C (7 days after birth) Sinus rhythm, [frequent](https://cn.bing.com/dict/search?q=Frequent&FORM=BDVSP6&cc=cn) [Ventricular](https://cn.bing.com/dict/search?q=Ventricular&FORM=BDVSP6&cc=cn) premature beat. D (9 days after birth) Sinus rhythm and occasional atrial premature beats. E Sinus rhythm

**Table 1.** Characteristics of the patient in time

| Parameters | D1 | D3 | D4 | D5 | D6 | D7 | D8 | D9 | D10 | M1 | M2 | M6 |
| --- | --- | --- | --- | --- | --- | --- | --- | --- | --- | --- | --- | --- |
| ECG | NSR | Ⅲ° AVB | Ⅲ° AVB | Ⅲ° AVB | NSR | NSR | NSR | NSR | NSR | NSR | NSR | NSR |
| Lowest HR (bpm) | 144 | 65 | 70 | 60 | 100 | 100 | 110 | 112 | 108 | 110 | 115 | 112 |
| Lowest BP (mmHg) | 78/50 | 76/44 | 55/28 | 60/41 | 80/58 | 88/50 | 94/59 | 89/52 | 87/49 | 90/50 | 93/52 | 91/49 |
| Lowest SpO_2_ (%) | 95 | 80 | 50 | 70 | 97 | 92 | 95 | 93 | 94 | 93 | 95 | 96 |
| Lowest PaO_2_ (mmHg) | 80 | 58 | 38 | 46 | 74 | 82 | 69 | 71 | 56 | 82 | 85 | 80 |
| SSA | / | / | +++ | +++ | +++ | +++ | +++ | +++ | +++ | +++ | ++ | + |
| SSB | / | / | +++ | +++ | +++ | +++ | +++ | +++ | +++ | ++ | + | + |
| RO52 | / | / | +++ | ++ | ++ | ++ | ++ | ++ | ++ | ++ | + | + |
| ANA | / | / | 1:320 | 1:100 | 1:100 | 1:100 | 1:100 | 1:100 | 1:100 | - | - | - |
| BNP (pg/ml) | / | >5000 | >4998 | >5000 | >5000 | >5000 | 1109.39 | 235.8 | 180.5 | 100.5 | 21.82 | 20.05 |
| CK-MB (µg/L) | / | 34.52 | / | / | / | / | 0.88 | / | / | 6.42 | / | / |
| Myoglobin (µg/L) | / | 135.2 | / | / | / | / | <21 | / | / | <21 | / | / |
| TnT-HSST (µg/L) | / | 4.48 | / | / | / | / | 0.037 | / | / | 0.094 | / | / |
| CK (U/L) | 450.4 | / | / | 129.64 | / | / | / | / | 140.28 | 120.7 | 72.9 | / |
| LVDd (mm) | / | 15 | 19 | 19 | / | 18 | / | 17 | 17 | 16 | 17 | 20 |
| LVEF (%) | / | 68 | 64 | 45 | / | 66 | / | 70 | 65 | 68 | 69 | 65 |
| PAP (mmHg) | / | 19 | 53 | 41 | / | 22 | / | 22 | 26 | 20 | 25 |  |
| WBC (×10^9^/L) | 7.62 | 11.19 | 14.93 | 14.06 | 7.98 | 9.29 | 8.63 | 8.25 | 7.68 | 7.95 | 8.23 | 9.13 |
| RBC (×10^12^/L) | 5.67 | 3.31 | 3.58 | 14.62 | 3.18 | 4.22 | 4.05 | 4.11 | 4.05 | 3.85 | 4.13 | 4.66 |
| HGB (g/L) | 217 | 121 | 126 | 105 | 101 | 136 | 131 | 128 | 126 | 125 | 112 | 130 |
| PLT (×10^6^/L) | 198 | 148 | 146 | 181 | 138 | 162 | 116 | 124 | 132 | 158 | 163 | 195 |
| PCT (ng/ml) | 0.12 | 1.4 | / | / | / | 0.17 | 0.07 | 0.06 | 0.05 | 0.05 | 0.03 | / |
| CRP (mg/L) | <0.5 | 0.86 | 1.79 | 1.34 | 0.49 | <0.5 | <0.5 | <0.5 | <0.5 | <0.5 | <0.5 | <0.5 |
| C3 (g/l) | / | 0.49 | / | 0.55 | 0.54 | / | 0.68 | / | / | 0.90 | 0.84 | 0.81 |
| C4 (g/l) | / | 0.13 | / | 0.12 | 0.15 | / | 0.14 | / | / | 0.19 | 0.17 | 0.20 |
| pH | 7.493 | 7.48 | 7.45 | 7.43 | 7.42 | 7.49 | 7.38 | 7.39 | 7.38 | 7.40 | 7.42 | 7.44 |
| PO_2_ (mmHg) | 83 | 38 | 34 | 37 | 126 | 71 | 56 | 86 | 92 | 95 | 102 | 88 |
| Lac (mmol/L) | / | 8.2 | 5.1 | 7.3 | 7.0 | 4.5 | 2.3 | 2.1 | 1.8 | 1.0 | 0 | 0 |

D1-D10: day of birth; M1-M6: months of birth; ECG: electrocardiogram; NSR: normal sinus rhythm; AVB: atrial ventricular block; HR: heart rate; BP: blood pressure; SpO_2_: oxygen saturation; PaO_2_: partial pressure of oxygen; ANA: antinuclear antibody; BNP: brain natriuretic peptide; CK-MB: creatinine kinase MB; TnT-HSST: troponin T-hypersensitivity; CK: creatinine kinase; LVDd: left ventricle end-diastolic diameter; LVEF: left ventricle ejection fraction: PAP: pulmonary artery pressure; WBC: white blood cells; RBC: red blood cells; HGB: hemoglobin; PLT: platelets; PCT: procalcitonin; CRP: C-reactive protein; C3: complement 3; C4; complement 4; PO_2_: partial pressure of oxygen; Lac: lactate; /: missing; -: negative.
