# Supplementary material for: Reverse complete heart block using transcutaneous pacing and repeated plasmapheresis in a neonate with lupus: a case report
Source: Pediatr Rheumatol Online J. 2023 Nov 9;21:135. doi: 10.1186/s12969-023-00920-w (PMC10636865; doi:10.1186/s12969-023-00920-w)
Supplement: Supplementary file 2 — Supplementary Material 2 [file 12969_2023_920_MOESM2_ESM.docx]

Dear Editor,

Thank you for carefully reviewing our manuscript previously titled “Reverse complete heart block using transcutaneous pacing and repeated plasmapheresis in a neonate with lupus: A case report” for possible publication in the Pediatric Rheumatology. We are grateful to you and your reviewers for their constructive critique. We have revised the manuscript, highlighting our revisions in red. and have attached point-by-point responses detailing how we have revised the manuscript in response to the reviewers' comments below.

Thank you for your consideration and further review of our manuscript. Please do not hesitate to contact us with any further questions or recommendations.

Yours Sincerely,

Prof. Zhangxue Hu,

Department of Pediatrics & Neonatology, Army Medical Center, Army Medical University, Chongqing, China, 400042

No.10 Changjiangzhilu, Yuzhong district, Chongqing, China, 400042

Email: [huzx1@163.com](mailto:huzx1@163.com)

Tel: 8618696668598

Reviewer Comments:
**Reviewer #1:**

I do not prefer of applying this medical management in all NL-CHB. The risk of plasmopharesis should be considered before deciding of using it as part of CHB management.

**Response**: Thanks to your valuable suggestions. We also agree that this medical management is not suitable for all NL-CHB. Fortunately, when the plasmopharesis was firstly applied in the treatment of the NL-CHB patient, who face that the initial antibody attack occurred not too long before birth and the neonate is still in the actionable window, significant therapeutic effect was observed. The normal sinus rhythm was successfully reconstructed, which the other NL-CHB treatments can not achieve this effect.

Before we conducted the plasmopharesis, the possible risks and treatments are listed below：

1. The possibility of Plasmapheresis can be implemented in treating NL-CHB. Plasmapheresis in neonates has no consensus on the absolute indication unlike adults and older children. Although plasmapheresis is an uncommon procedure in neonates and is fraught with technical challenges, it has been successfully performed in neonates[1,2,3]. Plasmapheresis in neonates and small infants is a treatment at the forefront used to effectively treat severe conditions, such as neonatal hyperbilirubinemia[4] , thrombotic microangiopathies[5], and acute liver failure[6]. Reports on the use of plasmapheresis in neonates are scarce.
2. Most adverse reactions are mild and clinically insignificant. As younger age was not a risk factor for adverse events in the plasmapheresis procedure, it is a complex process having various technical difficulties including problems related to vascular access, hemodynamic instability, bacterial infections, bleeding due to thrombocytopenia or other coagulation system dysfunction, hemodynamic instability in critically ill patients, anaphylactic and other allergic reactions to plasma infusion, acute volume overload, and hypocalcaemia related to the use of citrate as anticoagulant[7,8,9]. Despite the fact that severe life-threatening events have been reported, however, most adverse reactions are mild and clinically insignificant.
3. In this case report, the challenges with vascular access and complications from the procedure have been considered for using the therapeutic plasma exchange procedure. (1) First at all, using plasmapheresis in neonates is feasible, as long as attention is paid to several possible pitfalls and appropriate measures are taken to prevent complications. Gaining vascular access for plasmapheresis in neonates may be technically difficult and hazardous, so catheters have been inserted by the most experienced personnel available, to prevent repeated attempts that may cause large hematomas, thrombosis, infection, or pseudoaneurysm. Because of the need for both inlet and outflow routes, a large double-lumen catheter should be used, as in our case. If a very small vessel diameter precludes the use of such a catheter, two single-lumen catheters can be inserted in two vessels. (2) To prevent hemodynamic complications, extracorporeal circulating blood during plasmapheresis should not exceed 10% of the patient’s total blood volume. Hence, large volume, adult-size tubing is not appropriate for use in neonates who have a very small blood volume. so we used the specialized newborn tubing what was primed with exogenous cross-matched, leukocyte-depleted whole blood, with the appropriate hematocrit. Fresh frozen plasma (FFP) was used for replacement for the neonate. (3) Finally, it was performed in the neonatal intensive care unit. It is crucial to closely monitor heart rate, blood pressure, respiratory rate, and arterial blood gas analysis during the procedure, to prevent and promptly respond to hemodynamic abnormalities and hypocalcaemia common in this case.

References:

1. LiKamWa A, Kobaitri K, Totapally BR. A Neonate With Cytokine Storm Managed With Steroids, Therapeutic Plasma Exchange, and Tocilizumab. Cureus. 2023;15(9):e45138.

2. Mathur G, Collins L, Tigges CR, Schlueter AJ. Solutions to technical challenges during therapeutic plasma exchange using the Spectra Optia on a 4 kilogram neonate. Transfus Apher Sci. 2018;57(2):201-203.

3. Jung S, Kang ES, Ki CS, Kim DW, Paik KH, Chang YS. Successful therapeutic plasma exchange in a 3.2-kg body weight neonate with atypical hemolytic uremic syndrome. J Clin Apher. 2011;26(3):162-5.

4. Bambauer R, el-Saadi R, Graf N, Jesberger HJ, Limbach HG, Cordes H. Plasmapheresis in newborns with hyperbilirubinemia. Artif Organs. 1992;16(5):472-6.

5. Paglialonga F, Schmitt CP, Shroff R, Vondrak K, Aufricht C, Watson AR, et al. Indications, technique, and outcome of therapeutic apheresis in European pediatric nephrology units. Pediatr Nephrol. 2015;30(1):103-11.

6. Schaefer B, Ujszaszi A, Schaefer S, Heckert KH, Schaefer F, Schmitt CP. Safety and efficacy of tandem hemodialysis and plasma exchange in children. Clin J Am Soc Nephrol. 2014;9(9):1563- 70.

7. Fateen T, Sultana N, Sarwar M, Saqlain N. Complications of Therapeutic Plasma Exchange in pediatric patients: An experience at a tertiary care hospital. Pak J Med Sci. 2023;39(4):994-998.

8. Brunetta Gavranić B, Bašić-Jukić N, Kes P. Therapeutic Plasma Exchange-Does Age Matter? A Single-Center Study. Artif Organs. 2016;40(8):786-92.

9. Pandey P, Setya D, Marik A, Ranjan S, Kumari S, Mandal S, et al. Single center experience of therapeutic plasma exchange in a resource-constrained setting: A study of trends in scope and complications. J Clin Apher. 2023;38(4):463-471.

**Reviewer #2:**

1. Is this the first case treated with plasmapheresis? If not, then it'll be informative to review and summarize the previous cases.

**Response**: Thanks to your valuable suggestions. We are extremely grateful to reviewer for pointing out this problem. we have added the suggested content to the manuscript.

**Modifications**: section Case presentation

Reports on the use of plasmapheresis in neonates are extremely scarce, reflecting the technical difficulties and potential risks in carrying out this procedure in this age group.This forefront treatment is effectively used to treat severe conditions in neonates, such as neonatal hyperbilirubinemia (9,10), acute kidney injury (11), and Hemolytic Uremic Syndrome (12). Sawyer et al. (13) examined the outcomes of a cohort of neonates with septic shock treated with plasmapheresis, and found that plasmapheresis can be performed in critically ill neonates with severe septic shock on extracorporeal life support and may be beneficial in select cases. LiKamWa et al. (14) have reported that a neonate with cytokine storm managed with plasmapheresis, steroids, and Tocilizumab, resulting in stable discharge of the baby. But to the best of our knowledge, the neonate described here is the first reported patient with NL-CHB to undergo plasmapheresis therapy.

2. The statement (lines 6-12, page 3) is a bit vague. What kind of autoimmune diseases? Please rephrase it.

**Response**: Thanks to your valuable suggestions. We deeply agree with the comment and re-wrote the sentence in the revised manuscript as the following.

**Modifications**: section Background

Nearly 1% of all pregnancies are complicated by maternal autoimmune disease, which include systemic lupus erythematosus (SLE), Sjögren syndrome, rheumatoid arthritis, mixed connective tissue disease, an undifferentiated autoimmune disease, or NL with cutaneous and/or cardiac manifestations in a previous pregnancy.

3. Non-standardized abbreviations at first require the full name. (NL-CHB), line 21, page 3. Line 51, page 5. What NSR?
   **Response**: Thanks to your valuable suggestions. We are very sorry for our negligence of standardized abbreviations.

NL-CHB is the abbreviation for neonatal lupus-complete heart block, NSR is the abbreviation for normal sinus rhythm. We added the full name and marked them in red in revised manuscript.

4. Is it relevant to highlight if the mother had antenatal care or not? Was fetal bradycardia detected during the antenatal follow-up?

**Response:** The mother had regular antenatal care including the fetal heart monitoring, but without treatment for her ten-year psoriasis. Meanwhile, the fetal bradycardia was not detected during the antenatal follow-up. We have added the description into the paper.

**Modifications:** section Case presentation

The mother had regular antenatal care including the fetal heart monitoring, but without treatment for her ten-year psoriasis. Meanwhile, the fetal bradycardia was not detected during the antenatal follow-up.

5. Line 43, page 3, were these autoantibodies prior to delivery? If it was before delivery, what was the indication to run maternal autoantibodies?

**Response:** These mother’s autoantibodies were detected prior to delivery, and the indication for conducting autoantibody testing was the presence of maternal ten-year psoriasis. We deeply agree with the comment and re-wrote the sentence in the revised manuscript as the following.

**Modifications:** section Case presentation

Prior to delivery, The mother’s serum antibodies were ANA (1:1000), SSA (+++), SSB (+++), and Ro-52 (+++).

6. Line 43, page 3: the myocardial enzyme spectrum, etc. Are you related to the patient or his mother? The statement is not clear. Please rewrite it clearly.

**Response:** Thanks to your valuable suggestions. The myocardial enzyme spectrum related to the patient.

**Modifications:** section Case presentation

The baby’s myocardial enzyme spectrum after admission indicated an increasing in creatine kinase-myocardial band isoenzyme (CK-MB).

7. I believe that integrating the legends into the text led to confusion! (e.g., Figure 1A, Lines 53-58, page 3.) This statement is not clear! "Deoxygenation [oxygen saturation (SpO2) <80%] with severe myocardial injury and acute heart failure [brain natriuretic peptide (BNP) >5000 pg/mL] were observed immediately and persisted".

Please cite the figures and refer to them with their legends.

**Response:** We are very sorry for our negligence on the expression of Figure 1A. Figure 1A displays the neonatal ECG results, and the levels of deoxygenation and brain natriuretic peptide which were shown in Table 1.

We have made correction according to the and re-wrote the sentence in the revised manuscript as the following.

**Modifications:** Case presentation

Electrocardiogram (ECG) monitoring showed a sudden onset of heart rate fall, P (142bpm) and QRS (78bpm) were isolated (ⅢAVB), ventricular escape beat, incomplete right bundle branch block, with ST-T change at 3 days after birth (Figure 1A). Addtionally, deoxygenation [oxygen saturation (SpO2) <80%] with severe myocardial injury and acute heart failure [brain natriuretic peptide (BNP) >5000 pg/mL] were observed immediately and persisted.

Special thanks to you for your good comments.

**Reviewer #3:**

Well written case report on successful treatment of complete heart block in neonate lupus by transcutaneous pacing and repeated plasmapheresis.

**Response:** Special thanks to you for your good comments.

**Reviewer #5:**

Thank you for the opportunity to review the manuscript Reverse complete heart block using transcutaneous pacing and repeated plasmapheresis in a neonate with lupus: A case report Regarding the same I have the following comments.
1. Were other causes of the patient's manifestations such as myocarditis ruled out?
2. Can the lesions at the brain level be attributed to the presence of antibodies in the patient?

**Response:** We are extremely grateful to reviewer for pointing out this problem.

1.The patient’s test revealed the patent foramen ovale by echocardiogram, normal CK-MB levels, and no evidence of viral infection. Furthermore, myocardial damage was produced following atrioventricular block. The diagnosis of myocarditis was dismissed for lack of sufficient evidence.

2.Limitations on the number of our patient, we haven't been able to clarify the relationship between the presence of antibodies and brain damage. Neurologic manifestations have been described (1). In one study, 7 of 87 infants exposed to maternal Ro/SSA were reported to have hydrocephalus, and 10 had macrocephaly (2). However, these findings have not been reported in other cohorts of anti-Ro/SSA-exposed children. Therefore, whether there is an association with anti-Ro/SSA antibodies or not is still uncertain.

Special thanks to you for your good comment.

References

Silverman E, Jaeggi E. Non-cardiac manifestations of neonatal lupus erythematosus. Scand J Immunol. 2010;72(3):223-5.

Boros CA, Spence D, Blaser S, Silverman ED. Hydrocephalus and macrocephaly: new manifestations of neonatal lupus erythematosus. Arthritis Rheum. 2007;57(2):261-6.
